# Diagnostic performance and problem analysis of commercial tuberculosis antibody detection kits in China

**DOI:** 10.1186/s40779-018-0157-6

**Published:** 2018-03-22

**Authors:** Xue-Juan Bai, You-Rong Yang, Jian-Qin Liang, Hui-Ru An, Jie Wang, Yan-Bo Ling, Zhong-Yuan Wang, Xue-Qiong Wu

**Affiliations:** 1 Army Tuberculosis Prevention and Control Key Laboratory, Beijing Key Laboratory of New Techniques of Tuberculosis Diagnosis and Treatment, Institute of Tuberculosis Research, 309 Hospital of Chinese PLA, Beijing, 100091 China; 2 Tuberculosis Department No.1. Institute of Tuberculosis Research, 309 hospital of Chinese PLA, Beijing, 100091 China; 3 Tuberculosis Department No.2. Institute of Tuberculosis Research, 309 hospital of Chinese PLA, Beijing, 100091 China; 4 Tuberculosis Department No.3, Institute of Tuberculosis Research309 Hospital of Chinese PLA, Beijing, 100091 China

**Keywords:** Tuberculosis, Tuberculosis antibody test, Smear, Culture

## Abstract

**Background:**

The diagnosis of bacterium-negative pulmonary tuberculosis (TB) and extra-pulmonary TB is challenging clinically. The detection of the anti-TB antibody has an important, auxiliary, clinical diagnostic value. Therefore, TB antibody detection kits should be screened and evaluated, and the reagents with the highest sensitivity and specificity should be chosen and used clinically.

**Methods:**

The diagnostic performance of 7 commercially available TB antibody detection kits (kits A, B, C, D, E, F and G) based on the gold immunoassay detection of immunoglobulin (Ig) G or IgM antibodies were simultaneously evaluated and compared in 62 TB cases and 56 non-TB cases in a laboratory. A retrospective analysis including 2549 cases was carried out to assess the clinical diagnosis values of bacteriological examinations and TB antibody tests (kits B and H used in the clinic).

**Results:**

The sensitivities of TB antibody kits A, B, C, D, E, F and G in the sera from 62 TB patients were 50.0%, 83.9%, 38.7%, 9.7%, 48.4%, 69.4% and 79.0%, respectively; the sensitivities in the sera from 24 smear-negative TB patients were 29.2%, 79.2%, 29.2%, 12.5%, 29.2%, 54.2% and 79.2%, respectively; the specificities in the sera from 56 non-TB patients were 73.2%, 25.0%, 85.7%, 96.4%, 78.6%, 78.6% and 50.0%, respectively. Of the 2549 clinically diagnosed cases, there were 1752 pulmonary TB cases, 505 extra-pulmonary TB cases, 87 old pulmonary TB cases and 205 non-TB cases. The positive results for smear, culture, TB antibody kit B and kit H in pulmonary TB cases were 39.8% (543/1365), 48.6% (372/765), 45.8% (802/1752) and 25.2% (442/1752), respectively; the results in extra-pulmonary TB cases were 3.4% (6/178), 5.8% (4/69), 35.4% (179/505), and 11.3% (57/505), respectively; the results in old pulmonary TB cases were 0% (0/64), 0% (0/30), 32.2% (28/87), and 9.2% (8/87), respectively; and the results in non-TB cases were 0% (0/121), 0% (0/56), 21.5% (44/205), and 2.4% (5/205), respectively. Of 624 smear-positive and/or culture-positive pulmonary TB cases, the sensitivities of antibody test kits B and H were 53.0% and 36.4%, respectively. Of 901 smear-negative and/or culture-negative pulmonary TB cases, the sensitivities of antibody test kits B and H were 42.5% and 19.0%, respectively. The positive rate of antibody detection in the bacterium-positive pulmonary TB cases was significantly higher than that in the bacterium-negative pulmonary TB cases (*P* < 0.05).

**Conclusions:**

The colloidal gold-labeled TB antibody IgG detection assay is a simple, rapid and economical method that provides a better clinical auxiliary diagnosis value on TB, especially in smear-negative pulmonary TB and extra-pulmonary TB. The production, quality control, screening and evaluation of antibody detection kits are very important for its clinical application.

## Background

In 2015, the World Health Organization (WHO) reported that there were 10.4 million new TB cases and 1.4 million TB deaths worldwide [1]. The tuberculosis (TB) epidemic is very severe in China. The Fifth National Tuberculosis Epidemiological Survey in 2010 showed that the prevalence rate of the pulmonary TB in people aged 15 and older was 459 per 100,000 population. The prevalence rate of smear-positive pulmonary TB was 66 per 100,000 population, and the bacterium-positive pulmonary tuberculosis prevalence rate was 119 per 100,000 population. These results suggest that the majority of TB patients suffered from bacterium-negative pulmonary TB [2]. Clearly, the current clinical routine bacteriological examination methods (smear, mycobacterial culture and gene amplification) cannot satisfy the need for early diagnosis and differential diagnosis of bacterium-negative TB [3, 4]. The immunological examination methods can indirectly detect *Mycobacterium tuberculosis* (*M.tb*) infection based on the immune response of the host. The source of the blood sample is accessible, the operational process is simple and fast, and it has become an important mean for the auxiliary diagnosis of TB, especially bacterium-negative pulmonary TB, extra-pulmonary TB and child TB [3, 5, 6].

The current detection methods for cellular immune response, for example, tuberculin skin test (TST), interferon gamma (IFN-γ) release assays (IGRAs)] cannot differentiate a latent TB infection from an active TB [7, 8]. Due to a disorder in the immune function of the TB patient, the Th 1-type immune response of the TB patient gradually weakened, the Th 2-type immune response was enhanced, and the Th 1-type transfer to Th 2-type humoral immune response gradually increased [9]. The anti-TB antibody serum detection is generally not affected by a latent TB infection [10]. Therefore, the detection of anti-TB antibodies provides a higher diagnostic value in clinical practice and is also easier to be popularized [3, 11].

Although TB antibody detection in clinical practice has become a common method for TB diagnosis, and its diagnosis efficiency has had many reports in China and other countries, many commercial antibody test reagents with poor sensitivity and specificity cannot meet the clinical requirements for an accurate TB diagnosis [12, 13]. The WHO Expert Group on Sera-diagnostics in July 2011 issued related policies on TB serological detection [14], where they warned that commercial TB serological detection produced a large number of false-positive or false-negative results and provided inconsistent and imprecise estimates of sensitivity and specificity. The serological assays with low sensitivity will produce more false-negative results, and those with low specificity will produce more false-positive results. The high proportions of false-positive and false-negative results adversely impact patient safety. WHO policy urged to use the accurate microorganism examination or molecular detection methods recommended and to prohibit the use of inaccurate and unauthorized blood tests [14]. To clarify the problem, explore solutions, and guide clinical applications, this paper carried out research on laboratory evaluation and a retrospective study of clinical TB cases. We compared and evaluated the diagnostic performance of 7 commercially available TB antibody kits that are based on the gold immunoassay detection of IgG antibodies in 62 TB cases and 56 non-TB cases, which will provide an experimental basis for clinical choice. A retrospective analysis including 2549 cases was carried out to evaluate the clinical diagnosis values of bacteriological examinations and TB antibody tests.

## Methods

### Sera collection

All the serum samples were remaining specimens from clinical examinations. Sixty-two sera were obtained from the cases diagnosed as definite pulmonary TB, tuberculous pleurisy, tuberculous lymphadenitis, bronchial TB, etc., according to the “WS288-2008 diagnosis standard of pulmonary tuberculosis” [15] (including their TB history, symptoms, signs, etiological examination, imaging examination and treatment efficacy) in the TB Department No.1, 2, 3, Institute of Tuberculosis Research, 309 Hospital of Chinese PLA, Beijing, China from December 2016 to March 2017. Forty-two cases were male, 20 were female, and the mean age was 43.9±18.9 years. Fifty-six sera were from the cases diagnosed as definite non-TB patients, including cases of lung cancer, pneumonia, carcinomatous pleurisy, pulmonary infection, lymphoma, cerebral infarction, bronchial asthma, and bronchiectasis complicated with respiratory tract infection; 32 cases were male, and 24 cases were female, and the average age was 56.4±18.2 years.

### Specimen smear and culture

In accordance with the Chinese Laboratory Science Procedure of Diagnostic Bacteriology in Tuberculosis [16], some specimens including sputum, bronchoalveolar lavage fluid (BALF), pleural effusion, cerebrospinal fluid, biopsy tissue, and pus were smeared and examined by Ziehl-Neelsen acid-fast staining, where acid-fast bacilli were found and reported as positive results. Some specimens (including sputum, BALF, pleural effusion, cerebrospinal fluid, biopsy tissue and pus) were cultured at 37 °C by the MGIT BACTEC 960 mycobacterial culture system (Becton, Dickinson Company, USA). The cultures were smeared and examined by Ziehl-Neelsen acid-fast staining, where acid-fast bacilli were found and reported as positive results. The bacteria in the cultures were not found for 41 days and were thus reported as negative results.

### Serological antibody detection

Eight commercial rapid gold immunoassays kits were used for the detection of serological antibody IgG or IgM against *M.tb* antigens in this study. The various antigens (Table 1) were immobilized on the test spots or the line of a sample pad to trap serum antibodies. The test procedure was performed according to the instructions of the kits. If the test spots or lines were clear, it was identified as a positive reaction. Fuzzy spots or lines were designated suspect reactions, which were interpreted as negative.

**Table 1:** The antigens used, antibody types detected and detection methods of 8 commercial TB antibody test kits were shown

Number	*M.tb* antigens used	Antibody types detected	Assay used
Kit A	MTB11-38kD-MTB8-MTB48 recombinant proteins	IgG	Colloidal gold chromatography assay
Kit B	38 kD-16 kD recombinant protein	IgG	Colloidal gold chromatography assay
Kit C	38 kD-16 kD recombinant protein	IgG	Colloidal gold chromatography assay
Kit D	LAM	IgG	Colloidal gold chromatography assay
Kit E	LAM, 38 kD-16 kD-14 kD-16.8 kD recombinant proteins	IgG, IgM	Colloidal gold chromatography assay
Kit F	Purified 38 kD nature protein	IgG	Colloidal gold filtration assay
Kit G	LAM, 38 kD, 16 kD, MPT63 recombinant proteins	IgG	Colloidal gold filtration assay
Kit H	Mixed antigens (from Korea, the antigen ingredient is not shown)	IgG	Colloidal gold chromatography assay

### The source of cases retrospectively analyzed

Two thousand five hundred and forty-nine cases from 4 TB Departments at the Institute of Tuberculosis Research, 309 Hospital of Chinese PLA, Beijing, China from January to December 2016 were retrospectively analyzed. According to the definite clinical diagnoses, the cases were divided into 3 groups as follows: (1) pulmonary TB group: 1752 cases, male 1137 cases, female 615 cases, mean age 43.6 ± 19.9 years; (2) extra-pulmonary TB group: 505 cases, including tuberculous pleurisy, osteoarticular TB, lymph node TB, tuberculous cerebrospinal meningitis, tuberculous peritonitis, urinary TB, tuberculous polyserous inflammation, pelvic TB, tuberculous pericarditis, intestinal TB, chest wall TB, liver TB, epididymis TB, breast TB, male 289 cases, female 216 cases, mean age 39.5 ± 17.0 years; (3) old pulmonary TB group: 87 cases with a history of TB or old lesions in the lung but no current TB, male 49 cases, female 38 cases, mean age 54.1 ± 15.7 years; (4) non-TB group: 205 cases, including non-TB respiratory diseases (for example, lung cancer, lung abscess, pneumonia, chronic obstructive pulmonary disease, pneumoconiosis, bronchiectasis with infection, and pulmonary embolism) and extra-pulmonary non-TB diseases (bacterial pleurisy, viral meningitis, malignant pleurisy, urethritis, pericarditis, bone tumor, synovitis of the joint, peritoneal malignancy, pleural mesothelioma, lymphoma, chronic pancreatitis, liver neoplasms, digestive tract tumors, systemic lupus erythematosus, and bladder neoplasms), male 119 cases, female 86 cases, mean age 51.9 ± 18.1 years. These cases were retrospectively analyzed, including their clinical diagnoses and results of smear, mycobacteria liquid culture, and 2 TB antibody IgG tests using kits B and H.

### Data analysis

All data were recorded in an Excel spreadsheet. The statistical analyses were done using Statistical Product and Service Solutions (SPSS) 18.0, SPSS Inc. USA. The chi-square test was used to assess the difference between different rates. *P* < 0.05 was considered significant.

## Results

### The performance of 7 TB antibody test kits evaluated

Seven commercially available TB antibody test kits (kits A, B, C, D, E, F and G) in China were evaluated simultaneously by analyzing the sera from 62 cases of TB patients and 56 cases of non-TB patients in a laboratory (Table 2). Their sensitivities, specificities, positive predictive values, negative predictive values and consistent rates are shown in Table 3. Of the 62 TB patients, 32 cases (51.6%) showed a positive result on the sputum smear. The sensitivities of TB antibody detection in smear-positive TB patients on 6 TB antibody test kits (kits A, B, C, E, F, G) were higher than those in smear-negative TB patients, in which the differences were significant for kits A and E (*P* < 0.05), but not for kits B, C, F and G (*P* > 0.05).

**Table 2:** The results of TB antibody detection by 7 TB antibody test kits (*n* (%))

Group	Positive number (positive rate) of TB antibody						
	Kit A	Kit B	Kit C	Kit D	Kit E	Kit F	Kit G
TB group(*n* = 62)	31(50.0)	52(83.9)	24(38.7)	6(9.7)	30(48.4)	43(69.4)	49(79.0)
Smear-positive TB (*n* = 32)	22(68.8)^*^	28(87.5)	16(50.0)	3(9.4)	19(59.4)^*^	25(78.1)	29(90.6)
Smear-negative TB (*n* = 24)	7(29.2)	19(79.2)	7(29.2)	3(12.5)	7(29.2)	13(54.2)	19(79.2)
No smear results (*n* = 6)	–	–	–	–	–	–	–
Non-TB group (*n* = 56)	15(26.8)	42(75.0)	8(14.3)	2(3.6)	12(21.4)	12(21.4)	28(50.0)

-. No data; ^*^P < 0.05 compared with smear-negative TB

**Table 3:** The comparison of detection indexes by 7 TB antibody test kits (%)

Detection index	Kit A	Kit B	Kit C	Kit D	Kit E	Kit F	Kit G
Sensitivity	50.0^*^	83.9	38.7^*^	9.7^*^	48.4^*^	69.4	79.0
Specificity	73.2^#^	25.0^#^	85.7	96.4	78.6	78.6	50.0^#^
Positive predictive value	67.4	55.3	75.0	75.0	71.4	78.2	63.6
Negative predictive value	56.9	58.3	55.8	49.1	57.9	69.8	68.3
Consistent rate	61.0	55.9	61.0	50.8	62.7	73.7^#^	65.3

^*^P < 0.05 compared with kit B; ^#^*P* < 0.05 compared with kit D

### The clinical performance of 2 TB antibody test kits

Two thousand five hundred and forty-nine clinically diagnosed cases, including 1752 pulmonary TB cases, 505 extra-pulmonary TB cases, 87 cases with old pulmonary TB and 205 non-TB cases, were retrospectively analyzed. The analysis included their clinical diagnoses and the results of smear, mycobacteria liquid culture, and 2 TB antibody IgG tests (kits B and H, which have been used in the Clinical Departments of our institute for several years). The results are summarized in Tables 4 and 5. Of 1752 cases with pulmonary TB, the sputum smear rate (1365/1752, 77.9%) was significantly higher than the sputum culture rate (178/505, 43.7%) (*P* < 0.05), but the positive rate of the sputum culture (372/765, 48.6%) was significantly higher than that of the sputum smear (543/1365, 39.8%). The sensitivities of antibody test kits B and H were 45.8%, 25.2%, and the specificities were 78.5%, 97.6%, respectively. Of 505 cases with extra-pulmonary TB, the sensitivity of antibody test kit B (35.4%) was significantly higher than that of the smear (3.4%) and culture (5.8%) (*P* < 0.05). The sensitivity of antibody test kit H (11.3%) was significantly higher than that of the smear (3.4%) (*P* < 0.05), but it was not significantly higher than that of the culture (5.8%, *P* > 0.05). Of 87 cases with old pulmonary TB, the positive rates of kit B and kit H were 32.2% and 9.2%, respectively.

**Table 4:** The clinical performance of 2 TB antibody test kits (positive rate, %)

Group	Smear	Culture	Kit B	Kit H
Pulmonary TB group (*n* = 1752)	39.8(543/1365)	48.6(372/765)^*^	45.8(802/1752)	25.2(442/1752)
Extra-pulmonary TB group (*n* = 505)	3.4(6/178)	5.8(4/69)	35.4(179/505)^*#^	11.3(57/505)^*^
Old pulmonary TB group (*n* = 87)	0(0/64)	0(0/30)	32.2(28/87)	9.2(8/87)
Non-TB group (*n* = 205)	0(0/121)	0(0/56)	21.5(44/205)	2.4(5/205)

^*^P < 0.05 compared with smear; ^#^*P* < 0.05 compared with culture

**Table 5:** The relationship between bacteriological examination and antibody detection in 1525 pulmonary TB cases with the bacteriological examination results (n (%))

Bacteriological examination	Positive number (positive rate) of TB antibody	
	Kit B	Kit H
Smear		
(+)(*n* = 543)	291 (53.6)	206 (37.9)
(−)(*n* = 822)	341 (41.5)	152 (18.5)
Culture		
(+)(*n* = 372)	208 (55.9)	148 (39.8)
(−)(*n* = 393)	174 (44.3)	82 (20.9)
Bacterium		
(+)(*n* = 624)	331 (53.0)^*^	227 (36.4)^*^
(−)(*n* = 901)	383 (42.5)	171 (19.0)

^*^P < 0.05 compared with bacterium (−)

Of 624 smear-positive and/or culture-positive (defined as bacterium-positive) pulmonary TB cases, the sensitivities of antibody test kits B and H were 53.0% and 36.4%, respectively. Of 901 smear-negative and/or culture-negative (defined as bacterium-negative) pulmonary TB cases, the sensitivities of antibody test kits B and H were 42.5% and 19.0%, respectively. The positive rates for antibody detection in the bacterium-positive pulmonary TB cases were significantly higher than those in the bacterium-negative pulmonary TB cases (*P* < 0.05, Table 5).

Of 901 cases with bacterium-negative pulmonary TB, the results of 2 TB antibody detection kits (B and H) are shown in Table 6; 15.2% (137/901) of cases had positive reactions in both kits. Combining the two kits increased the sensitivities from 42.5% and 19.0% to 46.3%, and their consistent rate was 68.9%.

**Table 6:** Comparison of the results of 2 TB antibody test kits in 901 cases of bacterium-negative pulmonary TB

Kit H	Kit B		
	+	–	Total
+	137	34	171
–	246	484	730
Total	383	518	901

## Discussion

The antigen induces B lymphocytes to produce the antibody, an immunoglobulin (Ig) with immune activity. Its main function is to produce an immune reaction with the antigen, blocking the pathopoiesis of the pathogen. The antibody is present in various specimens, for example in the serum, pleural effusion, cerebrospinal fluid, ascites, joint effusion, and urine. The serum is main samples detecting the antibodies, and it is also called serological detection. At present, although 3 main antibody types of IgG, IgM and IgA were detected for TB diagnosis [17], the antibody IgG accounts for 70–75% of the total serum. IgG plays an important role in the immune protection against infections, increases with disease severity and lasts for a long time [18]. The active TB patients usually had a higher level of IgG than of IgM and IgA [19]. IgG has been the main antibody type in clinical detection and had diagnostic value for active TB, especially for the diagnosis of bacterium-negative pulmonary TB [3, 19], extra-pulmonary TB [20] and TB in children [21], which were difficult to diagnose by etiological examinations.

At present, there are many commercial TB antibody kits utilized for clinical application, but their sensitivities and specificities for TB diagnosis varied greatly [3, 12, 13]. The performance of most kits could not meet the needs for clinical diagnosis. The WHO reported a laboratory evaluation of 19 rapid commercial TB serological tests and found different sensitivities (1% to 60%) and specificities (53% to 99%) in 2008 [12]. Steingart et al. [13] systemically reviewed and performed a meta-analysis of the research reports of commercial serum antibody test reagents from 1990 to 2010. The results showed that the sensitivities and specificities in 67 studies on active pulmonary TB with 5147 cases were 0% to 100% and 31% to 100%, respectively. In 25 studies on active extra-pulmonary TB with 1809 cases, the sensitivities and specificities were 0% to 100% and 59% to 100%, respectively. In this study, 8 commercial TB antibody kits were evaluated, and they also confirmed these differences (sensitivity 9.7%–83.9%, specificity 25.0%–96.4%). Of 7 kits evaluated at the same time, kits A, C, E and F all had an acceptable specificity (73.2%, 85.7%, 78.6%, 78.6%, respectively), but only kit F had a higher sensitivity (69.4%), thus making it a good choice for clinical use. Kit D had a very high specificity (96.4%) and the lowest sensitivity (9.7%), while kits B and G had the poorest specificity (25.0%, 50.0%, respectively) and a very high sensitivity (83.9%, 79.0%, respectively), these three kits are not suitable for clinical use. Of the 2 kits used in our institute, kit H had the highest specificity (97.6%) and a lower sensitivity (25.2%), and kit B had a middle specificity (78.5%) and a middle sensitivity (45.8%); however, the performance of the two batches of kit B was vastly different, which suggests that the quality of kit B is unreliable. The analysis of the possible reasons for the differences are shown as follows.

### Detection kit quality instability

Quality instability in detection kits is the main problem commercial reagents currently face; this instability makes them not ideal in clinical application. First, the quality of the antigen part purchased was not controllable. Second, the method of antigen preparation, its purity and the concentration of antigen used affected the quality of the detection reagents [22]. The sensitivity of TB antibody detection kits using a natural antigen is generally higher than those using recombinant proteins expressed in *Escherichia coli* [23]. For example, the sensitivity of kit D using a natural 38 kD antigen purified from *Mycobacterium tuberculosis* in this study was comparable to those of kits A, B, C, E, and G, which used a number of recombinant protein antigens. This result may be because most of the recombinant protein expressed in *Escherichia coli* was not soluble [24], and the space conformation of recombinant protein may change and then affect their reactivity with the antibody. For a highly sensitive and specific diagnostic result, it is important that the recombinant protein is highly pure and correctly folded. Low antigen purity easily leads to a cross-reaction with other bacteria, which causes a low specificity. An antigen concentration that is too low may result in low sensitivity, while too high of an antigen concentration may result in low specificity. Additionally, the high sensitivity of the kit may lead to a lower specificity, and the high specificity may lead to low sensitivity. Therefore, the manufacturer should reasonably control these two key evaluation indexes. The manufacturers cannot change the production process at will and should strictly control the quality of the reagent production. It is critical for the validity of the antibody tests that the clinical laboratory selects and evaluates the kits periodically.

### Different antigens selected

*M.tb* antigens of 8 TB antibody kits evaluated in this study were not quite the same (Table 1). The current studies found that the sera from active TB patients could only recognize approximately 10% of *M.tb* antigens, produce different levels of antibodies against varied *M.tb* antigens, and the same patients at different stages of the disease may also induce different immune response on different antigens [3, 9, 25]. With the development of the disease, *M.tb* proliferated and metabolized in vivo. The humoral immune response of the host will focus on the membrane-associated antigens to extracellular protein antigens [9], which cause a great difference among the type, number and level of the antigen recognized by each serum from TB patients. The individual differences in antigen recognition are the main characteristics of the human TB humoral immune response [25–27]. Kunnathvelayudhana et al. [9] detected TB antibodies in human sera through an *M.tb* proteome chip, where it found 13 active TB related proteins. Of the 8 kits in this study, 6 kits used an immunodominant 38 kD antigen, and 4 kits used an immunodominant 16 kD antigen. The highly reactive protein antigens usually contain many B-cell epitopes [28]. The membrane-associated protein may be derived from both viable and dead bacteria and may also come from macrophage-secreted exosomes, which may be recognized by the sera from the persons with latent TB infection and bacterium-negative TB patients and by the sera from the active TB patients and non-TB patients to result in false-positive results [9, 29]. If the *M.tb* antigen selected had a cross-reaction with the sera from a person infected by other bacteria, especially in the environment of non-TB *Mycobacteria*, it would be easy to obtain false-positive results [30]. Therefore, it is important to select a number of highly sensitive, strongly specific and complementary antigens to create mixtures or fusions to improve the performance of TB sera diagnosis [11, 31–33]. Additionally, the preparation method, purity and concentration of the antigen used also affects the quality of the kit [22]. In general, the sensitivity of the detecting antibody using a natural antigen is higher than that using *M.tb* recombinant protein expressed in *Escherichia coli* [23]. For example, kit D in this study used a natural purified 38 kD antigen. Its sensitivity using this single antigen was similar to those using several recombinant protein antigens (kits A, B, C, E, and G), which may be because the majority of the recombinant protein antigens expressed in *Escherichia coli* were insoluble [24], and the improper protein folding may change their native conformation and affect the immune reaction. The poor purity of the antigen also results in cross-reactivity with other bacterial antigens and affects the detection specificity. Too low of an antigen concentration could affect detection sensitivity, while too high of an antigen concentration could affect detection specificity.

### Different burden and metabolic status of M.tb in vivo

After *M.tb* infection, it can be divided into the following two stages from a clinical perspective: latent TB infection and active TB. It is also divided into the following three stages from an *M.tb* metabolic perspective: dormant status (latent infection), proliferation status (active infection) and active tuberculosis [34, 35]. Recent studies showed that different *M.tb* strains infected and *M.tb* with different growth status maybe express various types of the proteins [36, 37]. The *M.tb* cell wall antigen and extracellular protein mainly induced the production of anti-TB antibodies, while most latency-related proteins did not induce antibody production in vivo. The antibody level was related with the progress of *M.tb* infection, bacterium burden and replication state [9, 38, 39]. Our studies [25, 40] and Zhang et al. [11] showed that the antibody level in bacterium-positive TB patients was significantly higher than that in bacterium-negative TB patients, and in TB patients, the level was significantly higher than that in persons with latent TB infection, the PPD-negative healthy population and persons with the BCG vaccination. The reports from Kunnathvelayudhan et al. [9] and Steingart et al. [13] confirmed that the bacterial load in vivo increased antibody reactivity. This may be because most antigens selected in the kits were extracellular proteins or cell wall antigens from proliferating *M.tb*. While the antigens of proliferating phage were less expressed in paucibacteria or not expressed in dormant bacteria, this generally produces a low level of antibodies or no antibodies at all [10, 41]. Therefore, the antibodies can be used as markers of active *M.tb* infection or the progression of TB [19, 42, 43]. A few studies showed that the antibody level was always related to bacterial load in TB patients [44, 45].

### The effect of old TB

A TB history and an abnormal chest X-ray in non-TB patients were some of the main causes of false-positives [9]. This study showed that some old TB cases (32.2%, 9.2%) could be detected the antibody. The study from Feng et al. [17] showed that antibody levels had no significant difference before chemotherapy and 1–6 months after chemotherapy. This finding indicates that the antibodies in healed TB patients exist for a long time, which may be because *M.tb* was not completely clear in some healed TB patients. The antibodies were still present in a small amount of *M.tb* strains with irregular proliferation [17, 46]. Therefore, the TB antibody could not be used to judge the efficacy of the TB treatment (such as improvement and cure), nor as a diagnostic marker of TB recurrence. However, some reports were inconsistent with our studies, and they showed a decrease in antibody levels after chemotherapy [47, 48].

### Different detection technique

Most of the TB antibody test kits used in China used a colloidal-gold label technique, and a few used ELISA methods. Eight commercially available kits in this study used a colloidal-gold label technique, where 3 kits (kits F, G, and H) used a colloidal gold dot immunofiltration assay method, and 5 kits (kits A, B, C, D, and E) used a rapid colloidal gold immunochromatographic assay method. The antibody colloidal gold test method is simple, fast, does not require special equipment, and is easy to use in clinics. However, this method has a lower sensitivity and poorer specificity than other semi-quantitative detection techniques, such as ELISA [25, 49], chemiluminescence labeling system, fluorescence labeling system [27], liquid crystal optical sensor, and indirect immuno-polymerase chain reaction (I-PCR) assay [50]. If fluid-phase immunoassays are routinely used in the clinic, it will show high sensitivity and specificity for the detection of antibodies.

The diagnosis of bacterium-negative pulmonary TB and extra-pulmonary TB is a difficult problem that needs to be solved in clinics. In this study of 1525 pulmonary TB cases having bacteriological examination results, 59.1% (901 cases) were smear-negative and culture-negative pulmonary TB, which is consistent with many reported (50–70%) [51]. Of 901 bacterium-negative pulmonary TB cases, the positive rates of antibody detection using kit B and kit H were 42.5% and 19.0%, respectively. Although the sensitivities of different antibody reagents had a great difference in the bacterium-negative pulmonary TB cases (10–90% by Steingart et al. [13], 12.5–79.2% in 7 kits of this study), these results suggest that an antibody test is useful for the assistant diagnosis of some bacterium-negative pulmonary TB in clinical practice. In our clinical laboratory, the combined application of 2 kits could improve diagnostic reliability and the detection rate of bacterium-negative pulmonary TB.

This study retrospectively analyzed the antibody test results of 505 extra-pulmonary TB cases. The sensitivities of 2 kits (kit B 35.4% and kit H 11.3%) were highly variable, which is consistent with previous reports [13]. However, the sensitivity was significantly higher than that of a traditional sputum smear (3.4%) and Mycobacterium culture (5.8%). Thus, the humoral immune response test of the host can make up for lack of bacteriological detection to decrease missed diagnosis of extra-pulmonary TB.

## Conclusions

In summary, many current commercial TB antibody detection products were of poor quality, although bad-quality products are used in clinical practice, the clinical value of the TB antibody marker cannot be denied. Serological detection remains a common, simple, fast, and valuable diagnostic method in China having a large proportion of bacterium-negative TB. In the future, it may be useful to screen a high-risk population [13]. This test may also be used for early diagnosis of the TB infection or for screening a high-risk population [2, 27, 28]. Strengthening quality supervision and reasonable clinical evaluation of TB antibody detection kits are very important in the clinical application. We also look forward to new TB antibody diagnostic products with high sensitivity and specificity to appear.

## Abbreviations

IFN-γ: Interferon gamma
IGRAs: IFN-γ release assays
*M.tb*: *M. tuberculosis*
TB: Tuberculosis
TST: Tuberculin skin test
WHO: World Health Organization
